# Screen use, health status, and self-perceived academic performance among Swedish dental and nursing students: an exploratory cross-sectional survey study

**DOI:** 10.1186/s12909-026-10445-x

**Published:** 2026-09-21

**Authors:** Nikolaos Christidis, Moa Lärfars, Parul Khattar, Damyana Markus, Giancarlo De la Torre Canales, Maria Christidis

**Affiliations:** 1 Division or oral Rehabilitation Department of Dental Medicine, Karolinska Institutet, Huddinge, SE-14104 Sweden; 2 Department of Nursing Science, Sophiahemmet University, Stockholm, SE-114 86 Sweden; 3 Department of Neurobiology, Care Sciences and Society, Karolinska Institutet, Huddinge, SE-14183 Sweden

**Keywords:** Academic performance, Digital media use, Mental health, Physical health, Screen time, Temporomandibular disorders

## Abstract

**Background:**

This study examined how screen use, physical and mental health, and 3-question temporomandibular disorders (3Q-TMD) screening status relate to self-perceived academic performance among Swedish undergraduate dental and nursing students.

**Methods:**

Ninety-one first-semester dental and nursing students completed a survey comprising sociodemographic items, estimated daily screen use by purpose, and twelve self-report instruments. Self-perceived academic performance was measured with the Academic Performance Scale (APS) and analyzed as a continuous score. Bivariate group comparisons between lower and higher self-perceived academic performance used the Mann-Whitney U test or Welch’s t-test with effect sizes, and the Holm-Bonferroni procedure to control the family-wise error rate within families of outcomes. The primary analysis was a hierarchical linear regression on the continuous APS score, entering demographics, then total screen time, then purpose-specific screen use, then a psychological symptom burden composite and 3Q-TMD screening status.

**Results:**

Total screen time was unrelated to academic performance in every analysis. Purpose-specific screen use added 19.0% of explained variance beyond total duration (F(3,81) = 6.78, *p* < 0.001), whereas total duration added 2.2% beyond purpose and was not significant (F(1,81) = 2.37, *p* = 0.127). In the full model (R2 = 0.341, adjusted R2 = 0.266), academic screen use was positively associated with performance (β = 0.426, *p* < 0.001), while work-related screen use (β = -0.299, *p* = 0.003) and psychological symptom burden (β = -0.361, *p* = 0.001) were negatively associated with performance. Students reporting lower performance also reported poorer health-related quality of life in five of eight RAND-36 domains and were more likely to have a positive 3Q-TMD screening result, although the latter association did not survive adjustment for oral parafunctional behavior.

**Conclusions:**

Self-perceived academic performance was associated with the purpose of screen use and with psychological symptom burden, but not with total screen-time duration.

**Supplementary Information:**

The online version contains supplementary material available at https://doi.org/10.1186/s12909-026-10445-x.

## Background

Academic performance refers to the measurable outcomes of students’ work within an educational context and is generally treated as a component of the broader construct of academic success, which additionally encompasses attainment of learning objectives, acquisition of skills and competencies, and student satisfaction [1, 2]. Academic performance is multidetermined, and students’ health is among its established determinants [3]. Physical health factors such as cardiorespiratory fitness, dietary intake and pain conditions have all been linked to academic outcomes in higher education [4–10].

Mental health is a further determinant of particular relevance in this setting. Depression, anxiety and psychological distress are highly prevalent among university students [11–13], partly reflecting the transition into higher education, which commonly entails relocation, greater independence, financial strain and adaptation to unfamiliar academic and social environments [14, 15]. Students in the health field carry the additional burden of clinical training, in which responsibility for real patients and fear of error are prominent stressors [16, 17]. Stress is correspondingly common in these programmes [18] and can directly impair attention, working memory and decision-making, thereby affecting engagement with learning material and performance in examinations [19].

Stress also frequently co-occurs with physical symptoms, including headache, muscle tension and orofacial pain [20, 21]. Temporomandibular disorders (TMD), which affect the temporomandibular joint, the masticatory muscles and surrounding tissues, are a common cause of chronic orofacial pain and have been consistently associated with psychosocial factors such as depression, somatization and stress [22–26]. Chronic pain may in turn impair concentration and sleep, although its direct relationship with academic performance remains insufficiently characterized [27, 28]. Regular physical activity, by contrast, is associated with better cardiometabolic health, improved mood and self-esteem, and lower stress and anxiety among university students [29–32].

University students are extensively exposed to digital screens for both study and leisure. Prolonged screen exposure may displace physical activity, social interaction and sleep, each of which supports health and learning [33, 34], and smartphone use in particular has been associated with poorer sleep quality, insomnia and daytime sleepiness [35, 36]. Prolonged exposure has also been linked to eye strain, headache, and neck and musculoskeletal discomfort [37, 38]. Much of this literature, however, treats screen use as an undifferentiated quantity of time. Emerging evidence suggests that the purpose of use may matter more than its duration, with educational use potentially supporting learning while passive or recreational use is more often associated with adverse outcomes; socioeconomic background, individual characteristics and environmental context may further modify these associations [39]. This proposition has rarely been tested directly, and purpose-specific screen use has seldom been examined together with physical health, mental health and orofacial pain within a single cohort, or in students enrolled in healthcare programmes, in whom academic, clinical and often occupational demands coincide.

The aim of this study was therefore to determine how screen use, physical and mental health, and 3-question-TMD (3Q-TMD) screening status relate to self-perceived academic performance among Swedish undergraduate dental and nursing students.

## Materials and methods

This study was approved by the Swedish Ethical Review Authority (dnr: 2025-02798-01). Before participating, all participants received verbal and written information about the study, including that participation was voluntary and that they could withdraw at any time providing no reason. Informed consent was obtained electronically before access to the questionnaire was granted; participants could not proceed without confirming their consent by ticking the designated consent box. Participants were also assured that all collected data would be treated confidentially and that anonymity was maintained.

The processing of personal data complied with the General Data Protection Regulation (GDPR). No directly identifying information was collected. All data were securely stored in REDCap, with access being restricted to members of the research team only. Confidentiality was ensured through secure data management and limited access procedures.

### Study design

The manuscript was prepared in accordance with the STROBE guidelines for reporting cross-sectional studies [40], Additional file 1. This exploratory cross-sectional survey study forms part of a larger longitudinal research project designed to follow the same cohort of students under similar conditions: over five years for dental students, and over three years for nursing students. However, the current analysis is based solely on data collected during the first semester of the participants’ first year of study.

Data were collected anonymously using a survey consisting of informed consent, background questions, items to assess estimated screen time, and self-report questionnaires. Survey items were presented primarily in Swedish. Validated Swedish versions were used when available; when no validated Swedish version was available, the original English-language version of the instrument was administered. No study-specific translation or cross-cultural adaptation was performed for these instruments.

The survey was pilot tested on a previous student cohort. Based on respondents’ feedback and suggestions, revisions were made to better reflect the respondent perspective and to shorten items considered excessively time-consuming.

The instruments were selected to capture complementary domains considered relevant to student health and academic functioning, including general health-related quality of life, psychological symptom burden, physical activity, sleep, digital media-related behavior, and pain- and TMD-related factors. Where possible, established instruments with available Swedish versions were prioritized. RAND-36 was selected to provide a broad assessment of perceived physical and mental health across several domains rather than focusing on a single health condition.

### Study recruitment and population

The survey was distributed to dental students at Karolinska Institutet and nursing students at Sophiahemmet University and contribution was voluntary. Recruitment and questionnaire administration were conducted by the authors and collaborating researchers through in-person distribution of QR codes linking to the online survey during teaching. For dental students, also through the programme’s Facebook pages, whereas for nursing students also through their teaching platform.

Inclusion criteria were being a first-year dental or nursing student and completion of the survey. Exclusion criteria were enrolment outside the first semester of the respective programmes or failure to complete the survey.

No formal sample size calculation was performed because this exploratory study represents the baseline analysis of an ongoing longitudinal cohort. The sample therefore reflects all eligible students who participated during the recruitment period.

### Data-collection

Data were collected using the Research Electronic Data Capture (REDCap) system [42, 43]. To ensure participant anonymity while also enabling linkage of data across multiple time points, each participant was assigned a unique identification code generated by REDCap. Only the principal investigator had access to the key linking participant identities to the study data.

### The survey construct

Screen time was assessed as estimated daily screen use in hours and categorized into social, academic/educational, and work-related usage. Participants also reported screen time for weekdays and weekends separately. Responses were recorded using nine predefined categories ranging from “0–1 hour” to “8 hours or more.”

For the purpose of data analysis, each response interval was converted to its midpoint value; for example, the interval “3–4 hours” was assigned a value of 3.5 h. The category “8 hours or more” was assigned a value of 8.5 h. For the questions regarding weekday and weekend screen time, total daily screen time was calculated as a weighted daily average, in which weekday and weekend-day estimates contribute in proportion to their frequency within the week:

$$\begin{aligned}&\mathrm{Total}\:\mathrm{daily}\:\mathrm{screen}\:\mathrm{time}\:=\\&(5\times\mathrm{weekday}\:\mathrm{hours}+2\times\mathrm{weekend-day}\:\mathrm{hours}) / 7\end{aligned}$$

### Academic performance scale (APS)

Self-perceived academic performance was assessed using the eight-item Academic Performance Scale (APS) in English, developed by Birchmeier, Grattan, Hornbacher, and McGregory at Saginaw Valley State University [44]. Items are rated on a five-point Likert scale ranging from Strongly Disagree [1] to Strongly Agree [5] and summed to produce a total score ranging from 8 to 40, with higher scores indicating better self-perceived academic performance. The original scale documentation reports an internal consistency of 0.89, a two-week test–retest reliability of 0.85, and satisfactory concurrent validity. According to the original scoring scheme, scores are classified as failing [8], poor [9–16], moderate [17–24], good [25–32], or excellent [33–40]. These categories were used only for descriptive and secondary analyses, whereas the continuous APS score was used as the primary outcome.

### International physical activity questionnaire (IPAQ)

Physical activity was assessed using the Swedish short form version of the IPAQ [45] consisting of seven questions regarding activities of different intensity: walking, sitting, moderate-intensity activities, and vigorous-intensity activities. Participants were asked to report on their physical activity in terms of both frequency (days per week) and duration (hours and minutes per day). Total physical activity scores were derived from the frequency and duration of reported activities, and participants were subsequently classified into three physical activity levels: *low*,* moderate*,* or high.*

### RAND-36 item health survey (RAND-36)

Health-related quality of life was assessed using the Swedish version of the RAND-36 questionnaire [46]. The questionnaire comprises 36 items covering eight health domains: physical functioning, role limitations due to physical health problems, role limitations due to emotional problems, energy/fatigue, emotional well-being, social functioning, pain, and general health perceptions. Response formats vary across domains. Scores were calculated according to standard RAND-36 scoring procedures and transformed to a 0-100 scale, with higher scores indicating better perceived health status. Domain scores were calculated by averaging the transformed item scores within each domain.

### Depression anxiety and stress scale (DASS-21)

Depression, anxiety and stress was measured using the Swedish version of the DASS-21-questionaire [47]. The instrument comprises 21 items distributed across three domains. Participants were asked to rate the extent to which each statement applied to their experiences during the preceding week using a four-point Likert scale ranging from 1 (“Did not apply to me at all”) to 4 (“Applied to me very much or most of the time”). Higher scores indicate greater severity of symptoms within the corresponding subscale.

### State-trait anxiety inventory (STAI)

Trait anxiety, how dispositional anxious a person is across time and situations, and state anxiety, how anxious a person is feeling at a particular moment, was measured using the Swedish version of the STAI-questionnaire [48]. The inventory is mainly used for the assessment of anxiety but also includes traits of depression. The STAI contains twenty statements, and the participants were asked to rate how well each statement applied to them on a 4-point Likert scale where 1 corresponds to “almost never” and 4 to “almost always”. The total score ranges from 20 to 80, with higher scores indicating greater anxiety.

### 3-Question TMD pain screener (3Q-TMD)

Temporomandibular disorder (TMD)-related symptoms were screened using the Swedish version of the 3Q-TMD [49], which is included in the Diagnostic Criteria for Temporomandibular Disorders (DC/TMD). The 3Q-TMD consists of three yes-or-no questions: “Do you have pain in your temple, face, jaw, or jaw joint once a week or more?”, “Do you have pain once a week or more when you open your mouth or chew?”, and “Does your jaw lock or become stuck once a week or more?”. A positive 3Q-TMD screening result was defined as an affirmative response to at least one of the three questions, whereas participants responding negatively to all three questions were classified as screen-negative.

### Oral behavior checklist (OBC)

Oral behaviors were assessed using the Swedish-language version of Oral Behavior Checklist (OBC) [41], a 21-item self-report instrument assessing the frequency of sleep- and waking-state oral behaviors during the preceding month. Two items assess sleep-related behaviors and 19 items assess waking-state behaviors. Items are scored from 0 to 4 according to frequency, yielding a total score ranging from 0 to 84, with higher scores indicating more frequent oral behaviors. The total OBC score was used as a continuous variable in the present analyses.

### Pain vigilance and awareness questionnaire (PVAQ)

Pain-related hypervigilance was assessed using the Swedish version of PVAQ [50]. The instrument is designed to measure the extent to which individuals attend to, monitor, and remain aware of pain. The questionnaire consists of eight items rated on a six-point Likert scale ranging from 0 (“Never”) to 5 (“Always”). The total score ranges from 0 to 40 and is calculated by summing responses across all items, with higher scores indicating greater pain-related hypervigilance.

### TAMPA scale of kinesiophobia (TSK)

Kinesiophobia, defined as fear of movement, was assessed using the Swedish version of the seventeen-item TSK [51]. The instrument evaluates the extent to which individuals avoid movement due to fear of pain or risk of injury. Participants were asked to score statements on a 4-point Likert scale (1 = strongly disagree, 4 = strongly agree). A total score ranging from 17 to 68 is obtained after reversing items 4, 8, 12 and 16. The higher the score, the higher the degree of kinesiophobia.

### Pittsburgh sleep quality inventory (PSQI)

Sleep quality during the past month was assessed using the Swedish version of PSQI [52]. The instrument consists of 19 items grouped into seven components: subjective sleep quality, sleep latency, sleep duration, habitual sleep efficiency, sleep disturbances, use of sleep medication, and daytime dysfunction. The component scores are summed to yield a global PSQI score ranging from 0 to 21, with higher scores indicating poorer sleep quality.

### Adult ADHD self-report scale-V1.1 screener (ASRS-v1.1)

Attention-deficit/hyperactivity disorder (ADHD) symptoms were screened using the Swedish version of the Adult ADHD Self-Report Scale Screener (ASRS-v1.1) [53]. The instrument consists of six items assessing the frequency of ADHD symptoms experienced during the preceding six months. Responses are rated on a five-point Likert scale ranging from 0 (“Never”) to 4 (“Very often”). Scoring was performed according to the standard ASRS-v1.1 screening procedure, where endorsement of four or more items above the established cut-off indicates a symptom profile consistent with an increased likelihood of ADHD.

### Mobile phone-related experiences questionnaire (CERM)

Problematic mobile phone use was assessed using the English version of CERM [54]. The instrument consists of 10 items addressing negative effects due to mobile phone usage including behavioral and social consequences, reduced activities, loss of control, and an intense desire to remain connected. Participants rated each item on a four-point Likert scale ranging from 1 (“Not at all”) to 4 (“Very much”). The total score ranges from 10 to 40, with scores of 10–15 indicating no problems with mobile phone use, 16–23 indicating occasional problems, and 24–40 indicating frequent problems with mobile phone use.

### Fear of missing out scale (FoMO)

Anxiety related to the fear of missing out on social experiences or information was assessed using the English version of the FoMO Scale [55]. The scale consists of ten statements, such as “I get worried when I find out my friends are having fun without me.” Responses are rated on a five-point Likert scale ranging from 1 (“Not at all true of me”) to 5 (“Extremely true of me”). A mean score is subsequently calculated to represent an individual’s level of FoMO, with higher scores indicating a stronger fear of missing out.

### Statistical analysis

Self-perceived academic performance, measured as the APS total score, was the outcome variable throughout. It was analyzed as a continuous score in the primary analysis; the four-band classification was retained only for descriptive purposes and for a secondary comparison between students with lower (Poor or Moderate, *n* = 19) and higher (Good or Excellent, *n* = 72) self-perceived academic performance. All instruments were scored across their full published range rather than reduced to severity categories, so that variance within categories was preserved. Continuous variables are presented as mean ± standard deviation and as median with interquartile range (IQR); categorical variables as frequencies and percentages.

Normality was assessed with the Shapiro-Wilk test and homogeneity of variance with Levene’s test, separately within each comparison group. Welch’s t-test was applied where both assumptions were met and the Mann-Whitney U test otherwise. Effect sizes are reported for every comparison as the rank-biserial correlation for Mann-Whitney U and as Cohen’s d for Welch’s t. Categorical variables were compared using the chi-square test, with Fisher’s exact test for two-by-two tables. For the between-group analyses, the Holm-Bonferroni procedure was applied separately within three pre-specified families of conceptually related outcomes: screen-use variables, RAND-36 domains, and psychosocial, sleep, oral behavior, and physical activity variables. Unadjusted and adjusted p-values are both reported.

Associations with the continuous APS score were first examined using Spearman rank correlations with 95% confidence intervals obtained by Fisher’s z transformation. For these correlation analyses, all 26 correlations with the APS score were treated as a single family and adjusted together using the Holm-Bonferroni procedure.

The primary analysis was a hierarchical linear regression predicting the continuous APS score. Predictors were entered in four blocks: (i) age, sex and programme; (ii) total daily screen time; (iii) social, academic and work-related screen use; and (iv) a psychological symptom burden composite and 3Q-TMD screening status. The block order was determined a priori to first account for demographic characteristics, then examine the contribution of total screen-time duration, followed by the additional contribution of purpose-specific screen use, which represented the central comparison of interest. Psychological symptom burden and 3Q-TMD screening status were entered in the final block to examine whether associations with screen use remained after adjustment for these health-related factors. The change in explained variance associated with block (iii) constitutes a direct test of whether the purpose of screen use accounts for variance beyond total duration; the reverse ordering was also fitted, entering total duration after purpose. To limit the number of correlated psychological predictors in the multivariable model, a psychological symptom burden composite was calculated as the mean of standardized scores for DASS-21 Depression, Anxiety and Stress, STAI-T, and ASRS-v1.1. The composite was used as an analytical summary measure rather than as a separate validated psychological construct and showed acceptable internal consistency in the present sample (Cronbach’s alpha = 0.833). Unstandardized coefficients with 95% confidence intervals, standardized coefficients and variance inflation factors are reported. Model assumptions were checked by inspection of residuals, the Shapiro-Wilk test, the Breusch-Pagan test, and leverage and Cook’s distance; the stability of the key increment in explained variance was assessed by bootstrap resampling with 4,000 replications.

A logistic regression was fitted with a positive 3Q-TMD screening as the outcome and APS, the symptom burden composite, sex, programme and oral behavior as predictors, in order to establish whether the bivariate association between positive 3Q-TMD screening results and academic performance persisted after adjustment. As a sensitivity analysis, the two extreme APS bands were compared directly, excluding the intermediate Good band, to provide a balanced contrast unaffected by the unequal group sizes of the primary comparison.

Analyses were performed in Python 3.12 with ordinary least squares and logistic regression implemented directly and verified against closed-form solutions. All tests were two-tailed, and the significance level was set at 0.05.

## Results

### Participants and academic performance

Of 230 eligible first-year students, 92 participated and 91 provided complete data, giving a sample of 41 dental and 50 nursing students. Eighty-one per cent were women, and the median age was 21 years (IQR 20–24). APS total scores ranged from 14 to 39 (mean 28.73 +- 5.49; median 29.00, IQR 26.00–32.00), with 3 students (3.3%) classified as Poor, 16 (17.6%) as Moderate, 51 (56.0%) as Good and 21 (23.1%) as Excellent. The two comparison self-academic performance groups did not differ in age, sex or programme (Table 1).

**Table 1:** Sociodemographic, educational and clinical characteristics (n (%)) of the study population according to self-perceived academic performance (*n* = 91)

	Groups		Between-group comparisons		
			Effect size	*p*-value	
	LAP (*n* = 19)	HAP (*n* = 72)	(Cramér’s V)	Fisher’s exact	Holm adjusted
*Programme*			0.00	0.802	1.000
Dentistry	8 (42.1)	33 (45.8)			
Nursing	11 (57.9)	39 (54.2)			
*Sex*			0.02	0.755	1.000
Men	3 (15.8)	15 (20.8)			
Women	16 (84.2)	57 (79.2)			
*Age*			0.03	0.615	1.000
≤ 21 years	11 (57.9)	36 (50.7)			
≥ 22 years	8 (42.1)	35 (49.3)			
*Previous work experience*			0.22	0.036	0.254
Yes	12 (63.2)	63 (87.5)			
No	7 (36.8)	9 (12.5)			
*Employed alongside studies*			0.12	0.272	1.000
No	11 (91.7)	45 (72.6)			
Yes	1 (8.3)	17 (27.4)			
*Financial stress*			0.10	0.301	1.000
None or low	6 (31.6)	34 (47.2)			
Moderate or high	13 (68.4)	38 (52.8)			
*Physical activity level (IPAQ)*			0.15	0.360	1.000
Low	3 (16.7)	10 (13.9)			
Moderate	11 (61.1)	33 (45.8)			
High	4 (22.2)	29 (40.3)			
*3Q-TMD screening*			0.24	0.018	0.143
Negative	6 (31.6)	46 (63.9)			
Positive	13 (68.4)	26 (36.1)			

*LAP* lower self-perceived academic performance (Academic Task Performance Scale band Poor or Moderate), *HAP* higher self-perceived academic performance (band Good or Excellent), *IPAQ* International Physical Activity Questionnaire, *3Q-TMD* 3-Question Temporomandibular Disorder Pain Screener. Age was dichotomized at the sample median. Financial stress was dichotomized as none or low versus moderate or high. Percentages are calculated within group. Employment data were unavailable for 17 participants and physical activity level for 1 participant. Holm adjustment applied across the eight comparisons

### Screen use

Total daily screen time did not differ between students reporting lower and higher self-perceived academic performance (median 5.21 vs. 5.43 h, *p* = 0.842), nor did social screen use (*p* = 0.207). In contrast, students with higher self-perceived academic performance reported approximately one additional hour of academic screen use per day (median 5.00 vs. 4.00, *p* = 0.023, *r* = 0.335), whereas those with lower self-perceived academic performance reported more work-related screen use (median 2.00 vs. 0.00, *p* = 0.018, *r* = -0.333). However, neither association remained significant after adjustment for multiple comparisons (Table 2).

**Table 2:** Median scores and median differences of screen-use variables according to self-perceived academic performance groups

	Groups		Between-group comparisons			
			Effect size		*p*-value	
	LAP (*n* = 19) median (IQR)	HAP (*n* = 72) median (IQR)	Median difference (95%CI)^*^	(*r*)	Mann-Whitney	Holm adjusted
Total screen time (h/day)*†*	5.21 (4.14–6.50)	5.43 (3.89–6.82)	0.00 (-1.00 to 1.00)	-0.05	0.842	0.842
Social screen use (h/day)	3.00 (2.25–3.75)	3.00 (2.00–4.00)	0.00 (-1.00 to 0.00)	-0.19	0.207	0.414
Academic screen use (h/day)	4.00 (3.00–5.00)	5.00 (4.00–6.00)	1.00 (0.00 to 2.00)	0.33	0.023	0.072
Work-related screen use (h/day)	2.00 (0.00–3.00)	0.00 (0.00–1.00)	-1.00 (-2.00 to 0.00)	-0.33	0.018	0.072

*LAP* lower self-perceived academic performance, *HAP* higher self-perceived academic performance, *IQR* interquartile range, *95%CI* 95% confidence interval, *r* rank-biserial correlation. *Total screen time was calculated as the weighted daily average (5 × weekday hours + 2 × weekend-day hours) / 7, and is therefore expressed on the same scale as the purpose-specific variables. †Welch’s t-test applied (both groups normally distributed with homogeneous variances); effect size is Cohen’s d. Hodges–Lehmann median difference: HAP minus LAP; positive values indicate higher scores in the HAP group. Purpose-specific screen use was reported for a typical day and total screen time separately for typical weekdays and weekend days; the two sets of items were not fully consistent, and the purpose-specific values sum to more than the reported total. Holm adjustment applied across the four comparisons

### Health-related quality of life

Students reporting higher self-perceived academic performance reported better health-related quality of life in five of the eight RAND-36 domains: role limitations due to physical health (*p* = 0.018), role limitations due to emotional problems (*p* = 0.007), energy and fatigue (*p* = 0.003), pain (*p* < 0.001) and general health (*p* = 0.035). After adjustment for multiple comparisons, the associations for role limitations due to physical health, role limitations due to emotional problems, energy/fatigue, and pain remained significant, whereas the association for general health did not (adjusted *p* = 0.139). No significant between-group differences were observed for physical functioning, emotional well-being, or social functioning (Table 3).

**Table 3:** Median scores and median differences of RAND-36 domain scores according to self-perceived academic performance groups

	Groups		Between-group comparisons			
			Effect size		*p*-value	
	LAP (*n* = 19) median (IQR)	HAP (*n* = 72) median (IQR)	Median difference (95%CI)^*^	(*r*)	Mann-Whitney	Holm adjusted
Physical functioning	95.00 (85.00–100.00)	100.00 (95.00–100.00)	0.00 (0.00 to 5.00)	0.19	0.176	0.527
Role limitations, *physical*	75.00 (50.00–100.00)	100.00 (75.00–100.00)	0.00 (0.00 to 25.00)	0.30	0.018	0.089
Role limitations, *emotional*	33.33 (0.00–50.00)	66.67 (33.33–100.00)	33.33 (0.00 to 66.67)	0.39	0.007	0.044
Energy/fatigue	30.00 (27.50–45.00)	50.00 (40.00–60.00)	15.00 (5.00 to 20.00)	0.44	0.003	0.024
Emotional well-being	56.00 (50.00–70.00)	64.00 (52.00–76.00)	4.00 (-4.00 to 12.00)	0.19	0.215	0.527
Social functioning	75.00 (56.25–87.50)	75.00 (62.50–87.50)	0.00 (-12.50 to 12.50)	0.04	0.800	0.800
Pain	77.50 (67.50–80.00)	90.00 (77.50–100.00)	20.00 (10.00 to 22.50)	0.48	< 0.001	0.006
General health	60.00 (47.50–65.00)	67.50 (55.00–80.00)	10.00 (0.00 to 20.00)	0.32	0.035	0.139

*LAP* lower self-perceived academic performance, *HAP* higher self-perceived academic performance, *IQR* interquartile range, *95%CI* 95% confidence interval, *r* rank-biserial correlation. All RAND-36 domains are scored 0–100, with higher scores indicating better perceived health. *Hodges–Lehmann median difference: HAP minus LAP; positive values indicate better health in the HAP group. Holm adjustment applied across the eight comparisons

### Psychosocial variables, sleep, oral behavior and physical activity

Students reporting lower self-perceived academic performance scored higher on trait anxiety (STAI-T; *p* = 0.002), on all three DASS-21 subscales (Depression *p* < 0.001, Anxiety *p* = 0.010, Stress *p* = 0.032), on the ADHD screener (*p* = 0.002), on problematic mobile phone use (CERM; *p* = 0.026) and on oral parafunctional behavior (OBC; *p* = 0.026). Trait anxiety, depression, anxiety and ADHD symptoms survived correction for multiple testing. Fear of missing out, kinesiophobia, pain vigilance, sleep quality and physical activity did not differ significantly between the groups (Table 4).

**Table 4:** Median scores and median differences of psychosocial, sleep and oral behavior variables according to self-perceived academic performance groups

	Groups		Between-group comparisons			
			Effect size		*p*-value	
	LAP (*n* = 19) median (IQR)	HAP (*n* = 72) median (IQR)	Median difference (95%CI)^*^	(*r*)	Mann-Whitney	Holm adjusted
STAI-T	52.00 (47.50–57.00)	45.50 (41.75–49.25)	-7.00 (-10.00 to -3.00)	-0.47	0.002	0.017
DASS-21 *depression*	16.00 (6.00–21.00)	4.00 (2.00–10.00)	-8.00 (-14.00 to -2.00)	-0.50	< 0.001	0.009
DASS-21 *anxiety*	8.00 (5.00–19.00)	6.00 (2.00–10.00)	-4.00 (-8.00 to -2.00)	-0.38	0.010	0.080
DASS-21 *stress*	18.00 (9.00–26.00)	12.00 (8.00–16.00)	-6.00 (-10.00 to 0.00)	-0.32	0.032	0.181
ASRS-v1.1†	12.00 (9.50–18.00)	8.00 (5.75–12.00)	-4.00 (-7.00 to -2.00)	-0.88	0.002	0.020
FoMO	19.00 (13.50–30.50)	18.50 (15.00–24.00)	-1.00 (-6.00 to 3.00)	-0.06	0.703	1.000
CERM	18.00 (16.00–23.50)	16.00 (14.00–18.25)	-2.00 (-5.00 to 0.00)	-0.33	0.026	0.181
OBC†	32.00 (22.00–40.00)	23.50 (16.00–32.25)	-7.00 (-14.00 to -1.00)	-0.72	0.026	0.181
TSK	30.00 (26.50–31.00)	27.50 (22.75–33.00)	-1.00 (-4.00 to 3.00)	-0.06	0.696	1.000
PVAQ†	19.00 (14.00–21.00)	19.00 (12.00–26.25)	1.00 (-4.00 to 6.00)	0.11	0.647	1.000
PSQI	7.00 (5.00–9.00)	6.00 (4.00–7.00)	-1.00 (-3.00 to 0.00)	-0.22	0.134	0.536

*LAP* lower self-perceived academic performance, *HAP* higher self-perceived academic performance, *IQR* interquartile range, *95%CI* 95% confidence interval, *r* rank-biserial correlation, *STAI-T* State-Trait Anxiety Inventory, trait version, *DASS-21* Depression Anxiety and Stress Scale, *ASRS-v1.1* Adult ADHD Self-Report Scale, *FoMO* Fear of Missing Out scale, *CERM* Mobile Phone-Related Experiences Questionnaire, *OBC* Oral Behavior Checklist, *TSK* Tampa Scale of Kinesiophobia, *PVAQ* Pain Vigilance and Awareness Questionnaire, *PSQI* Pittsburgh Sleep Quality Index. Higher scores indicate more symptoms on all instruments. *Hodges–Lehmann median difference: HAP minus LAP. †Welch’s t-test applied; effect size is Cohen’s d. Sleep quality data were unavailable for 1 participant. Physical activity is reported in Table 1. Holm adjustment applied across the eleven comparisons

### Association with the continuous APS score

At the bivariate level, the APS score correlated negatively with ADHD symptoms (rho = -0.397, *p* < 0.001), depressive symptoms (rho = -0.335, *p* = 0.001), problematic mobile phone use (rho = -0.265, *p* = 0.011), work-related screen use (rho = -0.254, *p* = 0.015) and trait anxiety (rho = -0.236, *p* = 0.024), and positively with academic screen use (rho = 0.269, *p* = 0.010), general health (rho = 0.240, *p* = 0.022) and role limitations due to emotional problems (rho = 0.229, *p* = 0.029). Total screen time was not associated with the APS score (rho = -0.069, *p* = 0.516). After correction across all 26 comparisons, only ADHD symptoms and depressive symptoms remained significant (Table 5). The psychological symptom burden composite correlated with the APS score at rho = -0.317 (*p* = 0.002).

**Table 5:** Spearman rank correlations between study variables and the Academic Performance Scale total score (*n* = 91)

		Correlation with APS score		*p*-value	
	*n*	rho	95%CI	Spearman	Holm adjusted
Academic screen use	91	0.27	0.07 to 0.45	0.010	0.238
Social screen use	90	-0.14	-0.34 to 0.07	0.183	1.000
Work-related screen use	91	-0.25	-0.44 to -0.05	0.015	0.333
Total screen time	91	-0.07	-0.27 to 0.14	0.516	1.000
RAND-36 physical functioning	91	0.12	-0.09 to 0.32	0.251	1.000
RAND-36 role limitations, *physical*	91	0.12	-0.09 to 0.31	0.274	1.000
RAND-36 role limitations, *emotional*	91	0.23	0.02 to 0.42	0.029	0.549
RAND-36 energy/fatigue	91	0.13	-0.07 to 0.33	0.203	1.000
RAND-36 emotional well-being	91	0.10	-0.11 to 0.30	0.355	1.000
RAND-36 social functioning	91	0.07	-0.14 to 0.27	0.507	1.000
RAND-36 pain	91	0.18	-0.03 to 0.37	0.097	1.000
RAND-36 general health	91	0.24	0.04 to 0.43	0.022	0.456
STAI-T	91	-0.24	-0.42 to -0.03	0.024	0.484
DASS-21 *depression*	91	-0.33	-0.51 to -0.14	0.001	0.029
DASS-21 *anxiety*	91	-0.12	-0.32 to 0.09	0.260	1.000
DASS-21 *stress*	91	-0.18	-0.37 to 0.02	0.083	1.000
ASRS-v1.1	91	-0.40	-0.56 to -0.21	< 0.001	0.003
FoMO	91	-0.07	-0.27 to 0.14	0.497	1.000
CERM	91	-0.27	-0.45 to -0.06	0.011	0.256
OBC	91	-0.11	-0.31 to 0.10	0.292	1.000
TSK	91	-0.13	-0.33 to 0.07	0.208	1.000
PVAQ	91	0.06	-0.15 to 0.26	0.577	1.000
PSQI	90	-0.02	-0.23 to 0.19	0.859	1.000
IPAQ (MET-min/week)	85	0.09	-0.12 to 0.30	0.403	1.000
3Q-TMD positive	91	-0.08	-0.28 to 0.13	0.434	1.000

*APS* Academic Performance Scale, *95%CI* 95% confidence interval obtained by Fisher’s z transformation. Positive values indicate that higher scores on the variable accompany higher self-perceived academic performance. Instrument abbreviations as in Tables 3 and 4. Holm adjustment applied across the twenty-six comparisons

### Hierarchical regression on self-reported academic performance

Age, sex and programme together explained 4.1% of the variance in APS scores and did not constitute a significant model (*p* = 0.31). Adding total daily screen time increased explained variance by 1.2%, which was not significant (F(1,84) = 1.06, *p* = 0.306). Adding the three purpose-specific screen-use variables increased explained variance by a further 19.0% (F(3,81) = 6.78, *p* < 0.001), and the model became significant overall (R2 = 0.243, *p* = 0.002). When the ordering was reversed and total screen time was entered after purpose-specific use, it added 2.2% of explained variance and remained non-significant (F(1,81) = 2.37, *p* = 0.127). Bootstrap resampling placed the 95% interval for the purpose-specific increment at 0.081 to 0.348.

In the final model (*n* = 89; R2 = 0.341, adjusted R2 = 0.266; F(9,79) = 4.55, *p* < 0.001), academic screen use was independently and positively associated with the APS score (b = 1.16, 95% CI 0.56 to 1.76; β = 0.426, *p* < 0.001), while work-related screen use (b = -0.91, 95% CI -1.51 to -0.32; β = -0.299, *p* = 0.003) and the psychological symptom burden composite (b = -2.63, 95% CI -4.15 to -1.10; β = -0.361, *p* = 0.001) were independently and negatively associated with it. Total screen time (*p* = 0.17), social screen use (*p* = 0.42), age (*p* = 0.51), programme (*p* = 0.27) and 3Q-TMD screening status (*p* = 0.28) were not associated with APS. Female sex was associated with a lower APS score after adjustment (b = -3.04, 95% CI -5.91 to -0.18; *p* = 0.038), although no such difference was present at the bivariate level. The maximum variance inflation factor was 1.65 and the maximum Cook’s distance 0.090, and excluding the seven observations exceeding the conventional influence threshold left all coefficients materially unchanged (Table 6).

**Table 6:** Hierarchical multivariable linear regression analysis of sociodemographic, screen-use and psychosocial variables associated with the academic performance scale total score

	b	95%CI	β	SE	*p*
*Model 1: Sociodemographic and programme*	*R² = 0.041; adjusted R² = 0.007; F(3, 85) = 1.20; p = 0.314*				
Age (years)	0.15	-0.05 to 0.34	0.17	0.10	0.132
Female sex (vs. male)	-0.08	-2.98 to 2.82	-0.01	1.46	0.956
Dentistry (vs. nursing)	1.86	-0.59 to 4.31	0.17	1.23	0.135
*Model 2: + total screen time*	*R² = 0.053; adjusted R² = 0.008; F(4, 84) = 1.17; p = 0.331*				
Age (years)	0.16	-0.03 to 0.36	0.19	0.10	0.100
Female sex (vs. male)	-0.19	-3.10 to 2.72	-0.01	1.46	0.898
Dentistry (vs. nursing)	2.14	-0.37 to 4.65	0.20	1.26	0.093
Total screen time (h/day)	-0.34	-0.98 to 0.31	-0.11	0.33	0.306
*Change in explained variance*	*ΔR² = 0.012; F(1, 84) = 1.06; p = 0.306*				
*Model 3: + purpose of screen use*	*R² = 0.243; adjusted R² = 0.177; F(7, 81) = 3.71; p = 0.002*				
Age (years)	0.05	-0.14 to 0.25	0.06	0.10	0.599
Female sex (vs. male)	-2.57	-5.44 to 0.30	-0.19	1.44	0.079
Dentistry (vs. nursing)	0.25	-2.22 to 2.71	0.02	1.24	0.843
Total screen time (h/day)	-0.50	-1.14 to 0.14	-0.17	0.32	0.127
Social screen use (h/day)	-0.32	-1.15 to 0.51	-0.08	0.42	0.444
Academic screen use (h/day)	0.95	0.32 to 1.57	0.35	0.31	0.003
Work-related screen use (h/day)	-0.97	-1.59 to -0.36	-0.32	0.31	0.002
*Change in explained variance*	*ΔR² = 0.190; F(3*,*81) = 6.78; p = < 0.001*				
*Model 4: + symptom burden and TMD*	*R² = 0.341; adjusted R² = 0.266; F(9*,*79) = 4.55; p = < 0.001*				
Age (years)	-0.07	-0.26 to 0.13	-0.08	0.10	0.507
Female sex (vs. male)	-3.04	-5.91 to -0.18	-0.23	1.44	0.038
Dentistry (vs. nursing)	-1.41	-3.94 to 1.11	-0.13	1.27	0.268
Total screen time (h/day)	-0.42	-1.03 to 0.19	-0.14	0.31	0.175
Social screen use (h/day)	-0.32	-1.11 to 0.47	-0.08	0.40	0.417
Academic screen use (h/day)	1.16	0.56 to 1.76	0.43	0.30	< 0.001
Work-related screen use (h/day)	-0.91	-1.51 to -0.32	-0.30	0.30	0.003
Psychological symptom burden composite (z)	-2.63	-4.15 to -1.10	-0.36	0.77	< 0.001
3Q-TMD positive (vs. negative)	1.24	-1.01 to 3.49	0.11	1.13	0.275
*Change in explained variance*	*ΔR² = 0.098; F(2, 79) = 5.90; p = 0.004*				

*b* unstandardized regression coefficient, *95%CI* 95% confidence interval, *β* standardized regression coefficient, *SE* standard error, *APS* Academic Performance Scale. The psychological symptom burden composite is the mean of standardized scores for DASS-21 depression, anxiety and stress, STAI-T and ASRS-v1.1 (Cronbach’s α = 0.833). Complete-case analysis (*n* = 89; 2 participants excluded for missing age or social screen-use data). Maximum variance inflation factor 1.65. Entering total screen time after purpose-specific screen use instead of before it yielded ΔR² = 0.022; F(1,81) = 2.37; *p* = 0.127

### 3Q-TMD screening results

A positive 3Q-TMD screening was more frequent among students reporting lower self-perceived academic performance (13 of 19, 68.4%) than among those reporting higher performance (26 of 72, 36.1%; chi-square = 5.16, *p* = 0.023; Fisher’s exact *p* = 0.018). In logistic regression, however, oral parafunctional behavior was the only independent predictor of a positive screen (OR 1.10 per OBC point, 95% CI 1.04 to 1.16, *p* = 0.001), while the APS score carried no independent association (OR 0.99, 95% CI 0.90 to 1.09, *p* = 0.81). Neither sex, programme nor the psychological symptom burden composite reached significance in that model (Nagelkerke R2 = 0.350). The Nagelkerke R² of 0.350 indicated that the full model provided substantially better explanatory fit than the intercept-only model, although this pseudo-R² should not be interpreted in the same way as the proportion of variance explained in linear regression.

### Sensitivity and subgroup analyses

Comparing the two extreme APS bands directly (Poor or Moderate, *n* = 19, versus Excellent, *n* = 21) reproduced the principal findings: academic screen use (*p* = 0.011), trait anxiety (*p* = 0.018), depressive symptoms (*p* = 0.004), ADHD symptoms (*p* < 0.001) and the psychological symptom burden composite (*p* = 0.008) all differed, while total screen time did not (*p* = 0.99); work-related screen use approached significance in the reduced sample (*p* = 0.052). Dental and nursing students did not differ in APS score (*p* = 0.26) or total screen time (*p* = 0.07); dental students reported more academic screen use (*p* = 0.016) and nursing students were older (*p* = 0.001) and reported more distress (*p* = 0.031) and more ADHD symptoms (*p* = 0.031). Work-related screen use did not differ between students who reported an ongoing job alongside their studies and those who did not (median 0.50 vs. 1.00 h, *p* = 0.70).

## Discussion

This study examined the associations between screen use, physical and mental health, and 3Q-TMD screening status with self-perceived academic performance among first-semester dental and nursing students. The principal finding was that the purpose of screen use, rather than its total duration, was associated with academic performance. Academic screen use was positively associated, whereas work-related screen use was negatively associated with academic performance. Both associations remained significant after adjustment for demographic characteristics, study programme, psychological symptom burden, and 3Q-TMD screening status. Psychological symptom burden also emerged as an independent correlate. Given the cross-sectional design and the reliance on self-reported measures, these findings should be interpreted as associations rather than causal effects.

Previous studies have reported an association between lower screen time and higher academic performance, either independently of physical activity [56] or in combination with it [57]. However, these studies consider screen exposure as a single undifferentiated construct. In the present sample, total screen time was not associated with academic performance across group comparisons, correlation analyses, and regression models. In contrast, disaggregating screen use according to its purpose revealed a substantial and statistically robust association. The marked difference in explained variance between purpose over duration (19.0%) and duration over purpose (2.2%) provides the strongest support from the present data for the conclusion that the context and function of digital media use, rather than the total amount of time spent using screens, are more closely associated with academic outcomes [37].

These findings also have methodological implications. Studies that measure only total screen time may produce null findings because of limitations in exposure measurement rather than the absence of a true association. Combining educational and recreational screen use into a single variable may obscure associations operating in opposite directions. Therefore, when screen exposure is investigated as a potential risk factor in educational or public health research, differentiating screen use according to its purpose appears essential rather than merely optional. The positive association between academic screen use and academic performance should not be interpreted as evidence that screen exposure enhances learning. Since academic performance was self-perceived, students who believe they study effectively may simply report spending more time engaged in academic activities, regardless of the medium used. Consequently, academic screen time should therefore be interpreted as an indicator of study engagement than as an independent determinant of academic performance.

Work-related screen use was also independently associated with academic performance, although its interpretation is less straightforward. An intuitive explanation would be that students combining paid employment with their studies have less time available for academic activities, experience greater stress, and have fewer opportunities for recovery [58], while additional screen-based work contributes to digital fatigue and cognitive overload [59]. However, this interpretation was not supported by the present data. Employment status was recorded as part of the survey, and work-related screen use did not differ between students who reported current employment and those who did not. Furthermore, students reporting lower academic performance were more likely to report having no previous work experience. Therefore, this variable does not appear to reflect employment in a straightforward manner. Alternative explanations remain possible. Participants may have interpreted the item as referring to time spent on administrative or other non-academic obligations, or it may capture a pattern of fragmented, obligation-driven screen use that displaces sustained studying. It is also possible that students with poorer perceived academic performance classified some of the same screen time as work rather than study, however, these possibilities cannot be distinguished using the present data. Thus, although the association was statistically robust, its underlying meaning remains uncertain. Clarifying the wording of this item and collecting direct measures of working hours should therefore be priorities in future follow-up assessments.

Psychological distress was independently associated with academic performance and represented the strongest bivariate correlate in the study. ADHD symptoms and depressive symptoms were the only variables that remained significant after correction across all 26 correlations. These findings are consistent with extensive evidence linking psychological distress to reduced academic achievement, delayed study progression, and lower academic self-efficacy [62, 63, 65], as well as systematic reviews demonstrating poorer academic outcomes among students with ADHD symptoms [60]. The relationship between ADHD symptoms and digital media use appears bidirectional and remains incompletely understood, with individuals reporting ADHD symptoms appearing more susceptible to problematic media use, while excessive media use may in turn influence symptom severity [61]. The observed association between problematic mobile phone use and lower academic performance is also consistent with previous evidence showing a negative association between problematic smartphone use and academic achievement [66].

A comparable pattern was observed for health-related quality of life. Students reporting lower academic performance also reported greater role limitations due to physical health and emotional problems, lower energy levels, increased bodily pain, and poorer general health perceptions. Together with the findings for psychological distress, these findings describe a coherent profile of strain associated with lower self-perceived academic performance rather than a series of isolated associations. Because of the cross-sectional design, the direction of these relationships cannot be established. Psychological distress is known to influence self-appraisal, and part of the observed association may reflect a shared negative response tendency across self-reported measures, including the outcome variable.

Positive 3Q-TMD screening results were more frequent among students reporting lower academic performance in the bivariate analyses, although this association was no longer significant after adjustment. In the logistic regression analysis, oral parafunctional behavior was the only independent predictor of a positive 3Q-TMD screening result, whereas academic performance showed no independent association. The most parsimonious interpretation is that the bivariate association was confounded by parafunctional behavior, which itself was more prevalent among students with lower academic performance, rather than reflecting a direct relationship between TMD and academic performance. This interpretation is consistent with previous studies among dental students reporting no association between TMD and academic performance [68, 69], as well as with the multifactorial aetiology of TMD, in which behavioral and psychological factors are well established [64, 67], whereas academic performance has not been established as such.

### Strengths and limitations

The principal strengths of this study include the use of established instruments scored across their full ranges, the inclusion of students from two healthcare programmes at two institutions at a comparable stage of education, and an analytical strategy that directly tested the central hypothesis rather than relying solely on bivariate associations. Reporting effect sizes and Holm-adjusted p-values alongside unadjusted p-values also enables readers to assess the robustness of each finding.

Several limitations should be acknowledged. First, all variables, including the outcome, were self-reported. Academic performance was assessed using the APS rather than objective academic records. Although related, these measures are not interchangeable, and shared method variance between the outcome and psychosocial predictors cannot be excluded. In addition, the APS, CERM, and FoMO instruments were administered in English because validated Swedish-language versions were not available. Although the original instruments were used without study-specific translation, measurement equivalence in Swedish first-semester students cannot be assumed, and this may have affected participants’ interpretation of individual items. This limitation is particularly relevant to the APS because it served as the primary outcome measure. Furthermore, screen-use variables were based on estimated hours reported using categorical response options rather than objectively recorded device data, making them susceptible to recall bias and the well-recognized discrepancy between self-reported and objectively measured screen time. The sample size was modest, and the comparison groups were unequally distributed, limiting statistical power. Consequently, non-significant findings for physical activity, sleep quality, kinesiophobia, and pain vigilance should not be interpreted as evidence of no association. Conversely, although the two screen-use variables did not remain significant after correction for multiple comparisons in the bivariate analyses, both were clearly significant in the multivariable regression model, which provides the principal support for their interpretation. With nine predictors and 89 complete cases, the final model approached the conventional recommendation regarding the number of observations per predictor. Although diagnostic procedures were satisfactory and bootstrap resampling supported the principal findings, replication in larger samples remains necessary.

Participation was voluntary, introducing the possibility of selection bias. Furthermore, the sample consisted exclusively of first-semester students from two Swedish institutions, limiting the generalizability of the findings to other populations and educational settings. Finally, data collection occurred at different points during the first semester and may therefore have coincided with periods of varying academic demands.

### Implications and future directions

For educators and student support services, the most relevant practical implication is that the total number of screen hours reported by students may provide limited information regarding academic difficulties, whereas the purpose of screen use appears substantially more informative. Consequently, recommendations focused solely on reducing overall screen time receive little support from the present findings. The co-occurrence of lower self-perceived academic performance with psychological distress, fatigue, pain, and positive 3Q-TMD screening results further suggests that academic and health support services should be integrated rather than delivered independently with psychological distress representing a particularly relevant target for assessment and support.

This survey represents the baseline assessment of a longitudinal project in which the same cohort will be followed annually throughout their education. To preserve longitudinal comparability, the same language versions of the instruments used at baseline will be retained in subsequent follow-up assessments. If validated Swedish versions become available, any transition would require careful consideration of measurement equivalence before implementation. Future assessments should clarify the wording and interpretation of the work-related screen-use item, directly measure working hours, incorporate objectively recorded screen-use data alongside self-reports, and, where ethically feasible, include documented academic records. Longitudinal follow-up will also allow investigation of temporal relationships that cannot be addressed using the present cross-sectional design, particularly whether psychological distress precedes or follows perceived academic underperformance.

## Conclusions

It can be concluded that self-perceived academic performance was associated with the purpose of screen use, but not with its total duration. Academic screen use was positively associated with performance, whereas work-related screen use and psychological distress were negatively associated, and these associations were independent of one another and of demographic characteristics, programme and 3Q-TMD screening status. The higher frequency of positive 3Q-TMD screening status among lower-performing students was explained by oral parafunctional behavior rather than by academic performance itself. These findings indicate that measuring total screen time is unlikely to be informative about academic performance and that the purpose of screen use, together with the psychological distress accompanying lower self-perceived performance, deserves closer attention in higher education.

## Supplementary Information

Additional File 1. The STROBE checklist. STROBE Statement - Checklist of items that should be included in reports of cross-sectional studies.

## Data Availability

The datasets generated and analyzed during the current study are not publicly available because data collection is ongoing as part of a longitudinal research project and public release could compromise future analyses and participant confidentiality. The datasets are available from the corresponding author upon reasonable request and subject to approval by the principal investigator and applicable ethical and data protection regulations.

## Abbreviations

SD: Standard deviation
TMD: Temporomandibular disorders
STAI: State-Trait Anxiety Inventory
DASS_D: Depression, Anxiety and Stress Scale, Depression
DASS_A: Depression, Anxiety and Stress Scale, Anxiety
DASS_S: Depression, Anxiety and Stress Scale, Stress
ADHD: Attention-deficit/Hyperactivity Disorder Scale
FOMO: Fear Of Missing Out
TSK: TAMPA Scale of Kinesiophobia
PVAQ: Pain Vigilance and Awareness Questionnaire
PSQI: Pittsburgh Sleep Quality Inventory
TMD: temporomandibular disorders
OBC: Oral behavior checklist
IPAQ: International Physical Activity Questionnaire
RAND-36: RAND 36-Item Short Form Health Survey
PF: Physical functioning
RP: Role limitations due to physical health
RE: Role limitations due to emotional problems
EF: Energy/Fatigue
EW: Emotional well-being
SF: Social functioning
P: Pain
GH: General health
IQR: Interquartile ranges
